# Gingival recession treatment with concentrated growth factor membrane: a comparative clinical trial

**DOI:** 10.1590/1678-7757-2019-0236

**Published:** 2020-03-27

**Authors:** Serap Karakış AKCAN, Berrin ÜNSAL

**Affiliations:** 1 Beykent University Faculty of Dentistry Department of Periodontology İstanbul Turkey Beykent University, Faculty of Dentistry, Department of Periodontology, İstanbul, Turkey.; 2 Gazi University Faculty of Dentistry Department of Periodontology Ankara Turkey Gazi University, Faculty of Dentistry, Department of Periodontology, Ankara, Turkey

**Keywords:** Blood platelets, Gingival recession, Plastic surgery, Tissue graft

## Abstract

**Objective:**

This clinical trial sought to evaluate the clinical effectiveness of concentrated growth factor (CGF) and compare it with connective tissue graft (CTG) with coronally advanced flap (CAF) in the treatment of Miller Class I gingival recessions (GR).

**Methodology:**

This split-mouth study included 74 Miller Class I isolated (24 teeth) or multiple (50 teeth) GRs in 23 jaws of 19 patients. GRs were randomly treated using CGF (test group: 37 teeth; 12 teeth in isolated GRs, 25 teeth in multiple GRs) or CTG with CAF (control group: 37 teeth;12 teeth isolated GRs, 25 teeth in multiple GRs). Clinical variables, plaque index (PI), gingival index (GI), probing depth (PD), recession depth (RD), recession width (RW), clinical attachment level (CAL), keratinized tissue thickness (KTT), keratinized tissue width (KTW), and root coverage (RC) were assessed at the baseline as well as at three and six months post-surgery. Healing index (HI) were obtained in the second and third weeks post-surgery. Postoperative pain was assessed for the first seven days using a horizontal visual analog scale (VAS).

**Results:**

No significant change was observed in PI, GI, or PD values in either the intergroup or the intragroup comparisons. A statistically significant decrease was observed in CAL, RD, and RW, and KTT increased in all groups at three and six months compared with the baseline. The control group had greater increases in KTW, KTT, and RC at three and six months. No significant difference was found in CAL or RD at the third and sixth months between the two groups. Healing was found to be similar for both groups in the second and third weeks post-surgery. The VAS values in the control group were higher than in the test group, especially at the second, fourth, fifth, and seventh days postoperatively.

**Conclusions:**

CTG is superior to CGF with CAF for increasing KTT, KTW, and RC. CGF may be preferable due to decreased postoperative pain.

## Introduction

Gingival recession (GR) is defined as the change in the position of the marginal part of the gums apically to the cement–enamel junction (CEJ) with denudation of the root surface. Gingival recession occurs because of anatomical, pathological, and traumatic factors.^1^

Numerous periodontal plastic surgery techniques have been suggested for treating GRs.^2^The connective tissue graft (CTG) with coronally advanced flap (CAF) procedure is the accepted gold standard for providing root coverage (RC), obtaining keratinized tissue gain and achieving predictable treatment outcomes.^2^However, this technique has several disadvantages, including insufficient donor tissue thickness, additional risk due to the presence of a second surgical site, extension of surgical procedure time, presence of a palatal neurovascular bundle in the proximity of the premolar–molar area, and limited graft size from the donor site with multiple defects or large recession areas. Furthermore, increased bleeding and pain complaints have been observed postoperatively. Therefore, alternative methods are used to treat GRs.^3,4^

Platelet concentrates (PCs) are used in the field of periodontology to provide key cells and growth factors to advance healing and promote regeneration.^5,6^ Platelet-rich fibrin (PRF) has shown to exhibit slow dissolution and a long-lasting fibrin network structure. This structure provides a matrix that contains high amounts of growth factors, thrombocytes, and leukocytes.^7^ Concentrated growth factor (CGF) is identified as a new approach to produce PRF or a next-generation PC.^8,9^ When producing CGF, the rotational speed of the centrifuge machine varies between 2400 and 3000 rpm.^8^ The variability of the rotation speed during centrifugation results in a fibrin matrix that is larger, more intensive, and includes more growth factors than PRF.^8-11^Some studies have reported that CGF induces osteogenic differentiation in periodontal ligament stem cells^9^and new bone formation for sinus augmentation,^10^ results in a defect fill that was found to be similar to collagen membranes in the surgical treatment of peri-implantitis,^12^ and has chemical and mechanical properties similar to those of advanced PRF (A-PRF).^13^ In the literature, only one clinical study has examined the treatment of multiple GRs with CGF. That study showed CGF with CAF surgery increased the keratinized tissue width (KTW) and keratinized tissue thickness (KTT) and may prevent postoperative relapse after the CAF procedure.^14^ No studies were conducted comparing CGF with CTG regarding clinical or patient-related parameters.

Therefore, this clinical study sought to evaluate the clinical efficacy of CGF combined with CAF in the treatment of GR and to compare their effectiveness with CTG. It also sought to assess and compare postoperative pain and soft tissue healing.

## Methodology

### Study population and design

This split-mouth, randomized, controlled clinical study protocol was approved by the Ankara University Faculty of Dentistry Clinical Research Ethics Committee, Ankara, Turkey (B.30.2.ANK.0.21.63.00/824-02/9-8/27;13/2, Clinical Trial.org-NCT03020732). Written consent was obtained from all patients before administration of treatment. A total of 19 patients were enrolled in this clinical trial. All were patients of the Gazi University Faculty of Dentistry, Department of Periodontology, and had aesthetic issues or cold/heat sensitivity problems associated with gingival recession; moreover, they all matched the features included in the criteria. This study was conducted from February 2013 to February 2014.

The inclusion criteria were: (1) at least 18 years of age; (2) systemically and periodontally healthy nonsmoker patients; (3) multiple adjacent or isolated Miller Class I GRs; (4) recession depth (RD) ≥2 mm, probing depth (PD) ≤3 mm, located on lateral, canine, or premolars on the same arch (maxilla or mandible); (5) identifiable CEJ; (6) absence of caries or restoration on the buccal surface and no previous history of endodontic problems or treatment; and (7) palatal donor tissue thickness ≥3 mm for CTG.

The exclusion criteria were: (1) patients having a smoking habit or systemic diseases that might contraindicate periodontal surgery; (2) use of medication affecting the blood clotting mechanism and wound healing; (3) previous periodontal surgeries in GR areas; (4) pregnancy, lactation, or oral contraceptive drug use for female patients; and (5) insufficient oral hygiene (full-mouth plaque and bleeding scores ≥15% after phase I periodontal treatment) or unchanged traumatic tooth-brushing habit.

All participants received phase I periodontal treatment one month before periodontal surgeries. Moreover, all participants underwent oral hygiene education and learned a non-traumatic tooth-brushing technique (the Roll technique) with an ultra-soft toothbrush as well as the use of dental floss or interdental brush for interproximal areas. Patients had either CAF with CGF (test) or CAF with CTG (control) surgeries on isolated (24 teeth on 9 maxillae and 3 mandibles) or multiple adjacent defects (50 teeth on 5 maxillae and 6 mandibles). A split-mouth design was used.

### Clinical measurements

All clinical measurements were performed by the same investigator (S. K. A.). A calibration protocol was applied to ensure the reliability of measurements. Five patients and 15 GRs were assessed regarding the KTW, KTT, PD, clinical attachment level (CAL), and RD parameters twice within 72 hours. Calibration was accepted when measurements were similar at the 90% level.^15^ Custom acrylic guides were prepared on patients’ plaster models for the standardization measurement. A periodontal probe (Nordent Manufacturing, Elk Grove Village, IL) was used to create mesio-buccal, mid-buccal, and disto-buccal reference notches on stents to obtain clinical data.

Plaque index (PI) and gingival index (GI) were recorded regarding the mean mesial, distal, and mid-facial surface measurement of a tooth.^16^ A periodontal probe was used at the mid-facial surface to obtain PD, CAL, RD, and KTW measurements; these measurements were rounded to the nearest millimeter. KTW was the distance from the free gingival margin to the mucogingival junction (MGJ), PD was the distance from the gingival margin to the bottom of the gingival crevice, and RD was the distance from the CEJ to the gingival margin. CAL was the distance from CEJ to the bottom of the gingival crevice. Recession width (RW) was measured as the horizontal distance between the mesial and distal recession borders coronally and parallel to the CEJ. The KTT value was obtained from a digital caliper (Stainless steel digital caliper, 150 mm, Insize, China) with an accuracy of 0.01 mm using a size 15 endodontic reamer on the mid-point localization of keratinized tissue at the level bottom of the gingival crevice.^15^RC was estimated using a formula.^17^All measurements were recorded at the baseline and repeated at three months and six months. Wound healing was scored clinically at two and three weeks according to a healing index (HI), when tissue color, bleeding at palpation, epithelization of incision margins, and presence of suppuration and granulation tissue were also evaluated. A score of 1 indicated very poor improvement and 5 indicated excellent recovery.^18,19^ The HI at 1 week postoperatively was not performed due to the removal of the periodontal dressing on the 14^th^ day after suture extraction. The Visual Analog Scale (VAS) with 100 mm was used to determine the patients’ postoperative pain level for the first seven days.^20^No pain was indicated as 0, and unbearable pain was indicated as 100. The patients received detailed information about the pain scale, and pain assessment was performed by the patients as self-reported. The patients were seen postoperatively on day 3 for the early recovery control, on day 7 for the delivery of the VAS scales and control of the periodontal dressing, on days 14 and 21 for the evaluation of HI, and in the first month for clinical measurements. Furthermore, additional appointments were scheduled at the third and sixth months.

### CGF preparation

**Figure 1:**
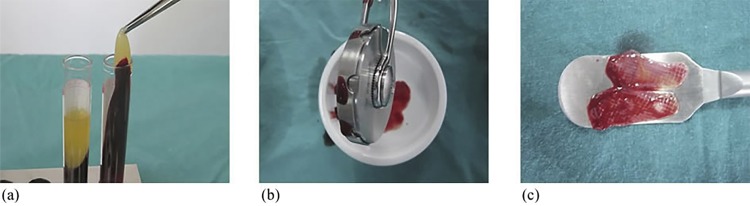
Concentrated growth factor (CGF). (a) Clots form and centrifuge tubes. (b) A special compressor. (c) CGF membranes

Immediately before the surgery test site, intravenous blood was drawn into two tubes without anticoagulant (Vacuette tubes, Greiner Bio-One North America Inc., USA). The blood was then centrifuged with a special centrifuge machine (Medifuge, Silfradent S.r.l., Sofia, Italy).^8^ The CGF was removed from the tube and separated from the red blood cell layer with a scissor; a special compressor was then used to obtain a CGF membrane of about 1 mm in thickness, as shown in Figure 1. Two CGF membranes were immediately placed into the recession area above the CEJ, in opposite directions.

### Surgical procedures

**Figure 2:**
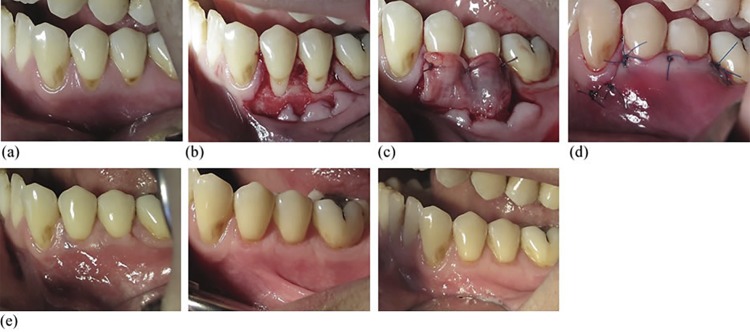
Test group. (a) Preoperative view. (b) Flap design and vertical incisions. (c) CGF placement. (d) CAF and sutures. (e) First, third and sixth months postoperative view. CGF. concentrated growth factor; CAF. coronally advanced flap

**Figure 3:**
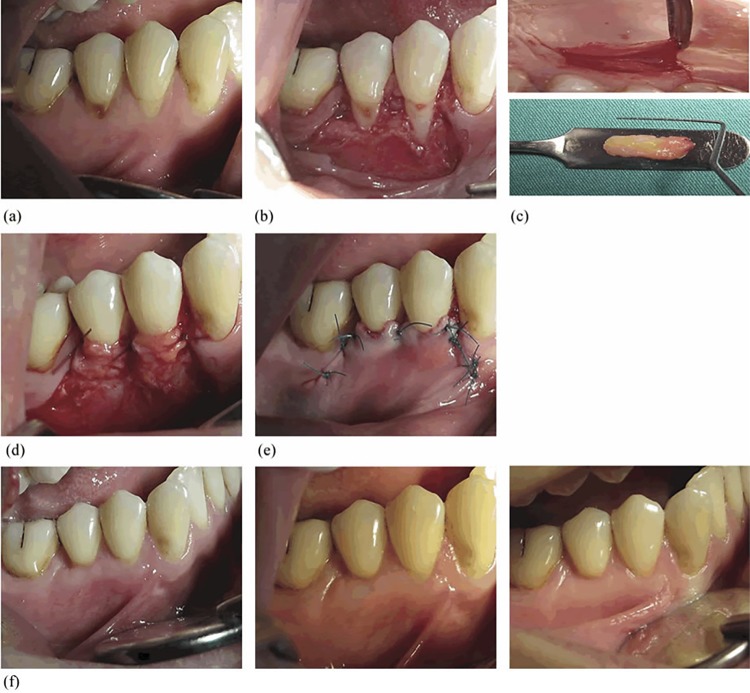
Control group. (a) Preoperative view. (b) Flap design and vertical incisions. (c) Palatinal area. (d) CTG placement. (e) CAF and sutures. (f) First, third and sixth months postoperative view. CTG. connective tissue graft; CAF. coronally advanced flap

All surgical procedures were performed in two separate sessions, one month apart, by the same surgeon (S. K. A.). Randomization was ensured with a coin toss by the same expert periodontist (B. Ü.) before the first surgeries. Local infiltration anesthesia (Ultracain D-S forte, Hoechst, Roussel, Frankfurt, Germany) was applied to the donor and the recipient sites. The technique, introduced by Langer and Langer^21^(1985), was similarly administered to prepare recipient sites. A 15-C blade (Swann-Morton Ltd., Sheffield, England) was used for incisions and sharp dissection, and all muscle attachments were removed apically from the MGJ. After papillae de-epithelization, either CTG or CGF was placed over the exposed root surface and fixed using suspension and simple, resorbable 5.0 sutures (Pegelak, Doğsan, İstanbul, Turkey). The flap was advanced 1 mm coronally from the CEJ to completely cover the CGF or CTG and was sutured with non-resorbable monofilament 5.0 sutures (Polyamid sutures, Seralon, Seragwiessner KG, Naila, Germany). This protocol was applied to both the test and the control groups, as shown in Figures 2 and 3.

CTG was taken from the area between the palatal canine teeth and the first molar teeth with a trapdoor technique. The graft horizontal and vertical sizes were adjusted to cover the open root surface and exceed 2 mm of adjacent bone margins. The graft thickness was adjusted between 1.5 and 2 mm, as shown in Figure 4C. The cover was placed back in position and fixed with silk 3-0 simple sutures (Doğsan, İstanbul, Turkey), as shown in Figures 2 and 3.

### Postoperative care

A periodontal dressing (Peripac, Dentsplay De Trey GmbH, Germany) on sterile aluminum foil was placed over the recipient area for both groups. The application of a cold compress extra-orally was recommended for the first 24 hours postoperatively, along with a soft-food diet and protection from trauma to surgical sites. Sutures were removed 14 days later, following which plaque control was achieved using an ultrasoft-bristle brush at the surgical areas. All patients were prescribed a nonsteroidal anti-inflammatory drug (Flurbiprofen, Majezik SR 200 mg, Sanovel, İstanbul, Turkey) for pain control, twice a day for five days, and a 0.12% chlorhexidine gluconate (Klorhex, Drogsan Pharmaceuticals, Ankara, Turkey) mouth rinse twice a day for two weeks. Antibiotics [amoxicillin with clavulanic acid (Augmentin) BID 1000 mg, GlaxoSmithKline] were also given twice a day for five days as a precaution against the risk of postoperative infection.

### Statistical analysis

The accepted sample size was 22 teeth for each group with a statistical power of 80%, according to the power analysis of other studies.^22^A total of 37 teeth were included in each group as a precaution against possible loss. In this study, the SPSS for Windows, version 22.0, (SPSS Inc., Chicago, IL, USA) was used for data analysis. The normality of the quantitative variables was investigated using the Kolmogorov–Smirnov test. Descriptive statistics such as mean ± standard deviation or median for continuous and discrete numeric variables (minimum–maximum) and demographic variables were shown for the number of cases and percentages. The Mann-Whitney U test was used for the intergroup evaluations. The Wilcoxon signed-rank test was used to assess changes in the double measurements, and the Friedman test was used to assess changes in the triple measurements (intragroup comparisons). The accepted significance levels were p<0.01 and p<0.05 for the results.

The primary outcome variable was the evaluation of median RC. The secondary clinical measurements were the PI, GI, PD, RD, RW, CAL, KTT, KTW, HI, and VAS pain scores.

## Results

A total of 74 Miller Class I GRs (24 isolated and 50 multiple defects) in 23 jaws (14 maxillae and 9 mandibles) of 19 patients (11 men and 8 women) were included in the study. The patients ranged in age from 20 to 63. All the participants completed the follow-up period of six months. In the maxillae, 38 GRs (22 premolars and 16 canines) and in the mandible, 36 GRs (22 premolars, 12 canines, and 2 lateral teeth) were treated. Two patients had multiple bilateral GRs in the maxilla and mandible.

The PI, GI, and PD median values were not significantly different in the intergroup and intragroup comparisons (p>0.05; Table 1). The CAL median values significantly decreased in both study groups at the third and sixth months when compared with the baseline (p<0.05; Table 1), and the control group had higher baseline CAL median values than the test group. The CAL values did not differ significantly between the groups (p>0.05; Table 1).

**Table 1:** Evaluation of PI, GI, PD and CAL clinical parameters and comparisons

Variable	Time	Control group(n=37)		Test group(n=37)		
		Mean±SD	Median-range	Mean±SD	Median-range	P^a^
PI	Baseline	0.02±0.07	0-0 (0)	0.03±0.09	0-0 (0)	0.623
	3 months	0.04±0.15	0-1 (0)	0.0±0.00	0-0 (0)	0.16
	6 months	0.03±0.09	0-0 (0)	0.08±0.18	0-1 (0)	0.889
**P**^**b**^			0.819		0.223	
GI	Baseline	0.07±0.20	0-1 (0)	0.06±0.12	0-0 (0)	0.79
	3 months	0.06±0.21	0-1 (0)	0.03±0.16	0-1 (0)	0.334
	6 months	0.04±0.13	0-1 (0)	0.07±0.19	0-1 (0)	0.643
**P**^**b**^			0.741		0.122	
PD	Baseline	1.43±0.55	1-3 (1)	1.39±0.54	1-3 (1)	0.711
	3 months	1.38±0.49	1-2 (1)	1.42±0.50	1-2 (1)	0.74
	6 months	1.46±0.55	1-3 (1)	1.36±0.54	1-3 (1)	0.406
**P**^**b**^			0.803		0.584	
CAL	Baseline	4.27±0.83	3-6 (4)	3.86±0.76	3-5 (4)	0.045*
	3 months	2.62±1.21	1-5 (3)	2.78±1.22	1-6 (2.50)	0.723
	6 months	2.54±1.01	1-5 (2)	2.56±1.08	1-5 (2)	0.963
**P**^**b**^			0.001**		0.001**	

^a^Mann Whitney U Test, statistically different between groups (*p<0.05).

^b^Friedman Test, significantly different compared with baseline (p<0.05, **p<0.01).

Regarding KTW and KTT, significant increases in the CTG group were observed between the baseline and other follow-up times (p<0.05; Table 1). The changes in the CGF group were statistically significant only for KTT; no significant difference was found for KTW. In the intergroup comparisons, statistically significant differences were observed at the third and sixth months in favor of the control group (p<0.05; Table 2).

**Table 2:** Evaluation of RD, RW, KTW, KTT and RC clinical parameters and comparisons

Variable	Time	Control group(n=37)		Test group(n=37)		
		Mean±SD	Median-range	Mean±SD	Median-range	P^a^
RD	Baseline	2.97±0.92	2-5 (3)	2.47±0.69	2-4 (2)	0.012*
	3 months	1.00±0.78	0-3 (1)	1.42±1.07	0-4 (1)	0.126
	6 months	0.92±0.82	0-3 (1)	1.22±0.92	1-2 (1)	0.139
**P**^**b**^			0.001**		0.001**	
RW	Baseline	3.54±0.65	2-5 (4)	3.39±0.76	2-5 (3)	0.304
	3 months	2.30±1.63	0-5 (3)	2.67±1.35	0-5 (3)	0.359
	6 months	2.16±1.67	0-5 (3)	2.44±1.36	0-5 (3)	0.565
**P**^**b**^			0.001**		0.001**	
KTW	Baseline	2.59±1.14	1-7 (2)	2.89±1.03	2-7 (3)	0.171
	3 months	3.49±1.07	2-8 (3)	2.92±1.02	2-7 (3)	0.004**
	6 months	3.57±1.14	2-8 (3)	3.03±1.02	2-7 (3)	0.019*
**P**^**b**^			0.001**		0.291	
KTT	Baseline	1.09±0.28	1-2 (1.06)	1.10±0.31	1-2 (1)	0.608
	3 months	1.69±0.30	1-2 (1.70)	1.43±0.32	1-2 (1.40)	0.001**
	6 months	1.63±0.31	1-2 (1.55)	1.38±0.34	1-2 (1.27)	0.001**
**P**^**b**^			0.001**		0.001**	
RC	3 months	66.60±26.29	0-100 (66.60)	50.45±28.79	0-100 (50)	0.015*
	6 months	72.45±22.92	33-100 (66.60)	52.54±33.97	0-100 (50)	0.006**
**P**^**c**^			0.038*		0.566	

^a^Mann Whitney U Test, statistically different between groups (**p<0.01, *p<0.05).

^b^Friedman Test, significantly different compared with baseline (**p<0.01).

^c^Wilcoxon Signed Rank Test, significantly different compared with baseline (*p<0.05).

The median RD and RW values decreased significantly between the baseline and other follow-up periods for both groups (p<0.05; Table 1), but they were not significantly different in the intergroup comparisons (p>0.05; Table 2). The CTG group had higher median RC values than the CGF group for all follow-up periods (p<0.05; Table 2). The increase in RC in the sixth month when compared with the third month was significant only in the control group (p<0.05; Table 2).

The median HI values significantly increased in both groups in the third week when compared with the second week (p<0.05; Table 3). In the intergroup comparisons, the median HI values were similar between the groups; without differences (p<0.05; Table 3). In the intragroup comparisons, the increase in the VAS median values seen in the following days was statistically significant in both groups when compared with the baseline (p<0.05; Table 3). The VAS median values of days 2, 4, 5, and 7 were significantly higher after CTG than after CGF (p<0.01; Table 3). No difference was observed for the other days.

**Table 3:** Postoperative pain and healing evaluations

Variable	Time	Control group(n=37)		Test group(n=37)		
		Mean±SD	Median-range	Mean±SD	Median-range	P^a^
VAS	Day 1	50.0±30.47	8-100 (45)	35.65±33.07	0-100 (24)	0.095
	Day 2	29.61±31.26	0-100 (20)	16.04±25.27	0-100 (4)	0.031*
	Day 3	19.74±29.35	0-100 (12)	10.78±24.52	0-100 (0)	0.065
	Day 4	22.65±30.75	0-100 (13)	4.57±12.72	0-56 (0)	0.005**
	Day 5	15.3±21.01	0-66 (0)	5.43±14.44	0-56 (0)	0.033*
	Day 6	11±21.31	0-65 (0)	4.91±13.61	0-50 (0)	0.166
	Day 7	11.09±19.07	0-60 (0)	3.61±12.84	0-57 (0)	0.041*
	**P**^**b**^		0.001**		0.001**	**P**^**c**^
HI	Week 2	3.86±0.69	3-5 (4)	3.78±0.73	2-5 (4)	0.779
	Week 3	4.34±0.71	3-5 (4)	4.26±0.61	3-5 (4)	0.56
	**P**^**d**^		0.002**		0.002**	

^a^Mann Whitney U Test, statistically different between groups (**p<0.01, *p<0.05).

^b^Friedman Test, significantly different compared with baseline (**p<0.01).

^c^Mann Whitney U Test, statistically different between groups (p<0.05).

^d^Wilcoxon Signed Rank Test, significantly different compared with baseline (**p<0.01).

A separate statistical evaluation was performed for the analysis of the difference in HI and VAS median values between the sites of isolated and multiple GRs, which were evaluated together in the test and control groups. According to this analysis, no significant difference was observed between the sites of isolated and multiple GRs in the test and control groups.

## Discussion

The effect of PC on the results of root coverage surgeries varies in the literature. The first reviews reported that PC treatments did not improve treatment results regarding RC, KTW, or CAL^23^, although recent reviews^24^ have reported that PRF reduces PD, attachment gain, RD, and patient morbidity. Recently, the changes in the centrifugation systems used for PC and the acquisition protocols have been believed to possibly change the properties of the fibrin structure and affect the clinical results.^25^ These new biomaterials are as follows: titanium-prepared PRF (T-PRF)^26^, A-PRF^25^, A-PRF+^25^, and CGF^8^. However, there are still too few clinical studies on the next generation of PRF-associated biomaterials in the literature. In this study, the CGF+CAF procedure was compared with CTG+CAF at the six-month follow-up. This is the first comparison in the literature.

The mean RC values for the CTG and CGF procedures were 72.45% and 52.45%, respectively. The CTG with CAF group was superior to the CGF with CAF group in terms of RC, KTT, and KTW. The treatment for the CGF with CAF group was found to be more effective only to reduce postoperative pain.

There are differing opinions as to whether techniques without vertical incisions provide better vascular support and healing^27^, aesthetic results^28^, positive effects on RC, and keratinized tissue gain^20^, or that vertical incision techniques provide better surgical vision, reduce flap tension^29^, shorten the operation time^30^ and provide RC.^31^ In this study, a technique with vertical incisions was preferred to provide better visibility and tension-free advancement of the flap for primary closure of the defect, CGF, and CTG.

CGF was first used by Doğan, et al.^14^ (2015) in the treatment of multiple gingival recessions in the maxilla. They reported that mean RC was 86.67% for the CAF (modified) with CGF group after six months. Doğan, et al.^14^(2015) reported that CGF treatment did not show any additive effect on the RC results of the CAF procedure. The authors^14^ suggested CGF application with CAF might increase the long-term stability of RC due to increasing KTW (0.58 mm) and KTT (0.32 mm), which caused a better attachment gain. Increases of 0.14 mm/0.28 mm, respectively, were observed in the KTW/KTT at the sixth month for the CGF group. Some results of this study showed increased KTT and attachment gains, which agrees with Doğan, et al.^14^ (2015). However, CGF had no effect on KTW and showed a lower RC percentage. The differences observed are possibly due to differences in the surgical and study designs. Unlike Doğan, et al.^14^ (2015), molar teeth were excluded in the study due to the RW values, larger surgical field, and complex anatomy. Another difference was that it included defects in both maxilla and mandible. Muscles in the maxilla and mandible have been reported to have different tensile strength and that differences in flap thicknesses in the jaws may produce different results on RC.^32^ This can be considered a limitation of this study.

In the literature, there are differences between the studies conducted with innovative PRF protocols and conventional methods, especially regarding the RC, KTT, and KTW results. PRF was suggested as an alternative to CTG for multiple and isolated GR.^3,22,32,33^ Uraz, et al.^32^ (2015) reported RC values as 95% and 96.1% after six months, and Tunalı, et al.^22^ (2015) as 76.63% and 77.3% after 12 months (22) for the PRF and CTG groups, respectively. PRF was superior regarding the increased KTT^15,29,33^ and postoperative patient comfort,^29,33^ whereas a higher KTW was reported for CTG^33^ in multiple GRs. The mean RC for T-PRF and CTG treatments were reported as 91.06% and 92.04%, respectively, at the sixth month, without difference between the groups.^27^T-PRF was reported as superior in terms of the resulting KTW, whereas CTG was found to be better than T-PRF for KTT in a recent study.^27^In this study, CGF did not seem to have improved clinical outcomes when compared with CTG. Although CGF membranes and others form a vital cell trap, their resorption times and stability may not be sufficient for the cells to repopulate in the region.^12^ In addition to clinical trial design and methodology differences in studies, the differences in centrifugation^25^ and the tube from which blood is collected^27^ may have affected clinical outcomes.^6^

Studies have suggested that PRF treatment increases KTW due to the release of growth factors involved in the stimulation and proliferation of gingival and periodontal fibroblasts.^3,4,18^ CTG includes connective tissue, which has the ability to induce keratinizing epithelium as a result of an increase in KTW.^3,4,18^Autogenous biomaterials are suggested to have functioned as gap fillers for an increase in KTT by increasing the tissue thickness such that it shows a histoconductive effect.^27^ Moreover, the increase in KTT is thought to be influenced by the initial flap/graft thickness and incisions.^3,27,33^ In this study, initial KTT and KTW values were similar. CTG thickness was applied at a thickness of 1.5–2 mm and CGF membranes were applied in 2 layers with an average thickness of 2 mm to eradicate a negative effect.

Culhaoglu, et al.^34^ (2018) reported they achieved a higher RC percentage when they compared a two-fold application of PRF with a four-fold application. RC values were 69.65% and 56.34% for 4PRF with CAF and 2PRF with CAF, respectively, at the sixth month.^34^ PRF was suggested to be administered as multilayered as possible.^34^ Similarly, in this study, a four-fold application of CGF could provide higher RC values. Another view is that placement of these autogenous biomaterials under the flap may limit the collateral circulation required for revascularization and healing.^3,33^ Comparative studies are needed to clarify the dose-dependent effect of PCs. Culhaoglu, et al.^34^ (2018) described the RD changes in the control group as “creeping attachment,” while the changes in the test group explained the degree of resorption in the membrane and the apical migration of flap.^34-36^ In this study, the control group had higher RD values at the baseline than the test group; however, no difference was observed at the sixth month. The higher RC values in the control group can be explained by the KTW increase and creeping attachment, in addition to the higher initial RD values. There was a significant decrease in CAL in both groups. Since the histological analysis was not performed, the type of attachment was not known.

PRF has been suggested to accelerate soft tissue healing with growth factors in addition to the fibrin network structure.^7,18,19^ Several studies have reported that PRF application decreases postoperative pain for the first seven days and accelerates healing during the first, second, and third weeks when compared with CTG or Emdogain.^18,19^ In this study, the median HI values were not different between test and control groups. Additionally, the CTG group had significantly greater VAS values than the CGF group. Postoperative discomfort and pain after CTG surgery are common, especially in the first week.^2^ Absence of donor site^15^ and reduction in operation time^33^ have been described to decrease the pain reported by the patient. The coexistence of single and multiple defects may have also affected the healing and pain evaluations. However, no difference was observed between these two groups according to the separate analysis for single and multiple defects.

Studies have reported that CTG size and thickness and their position in CEJ may negatively affect graft vascularization, flap dehiscence, RC, postoperative comfort, and aesthetics.^37,38^ Thinner, shorter grafts might provide better vascular nutrition from the flap covering the graft and connective tissue of the donor bed and might also improve postoperative comfort with aesthetic results.^37,38^ In this study, the CTG was standardized to be 1.5–2 mm thick, and the graft horizontal and vertical dimensions were adjusted to exceed 2 mm of the exposed root surface. Despite the attempt to establish a similar recipient bed environment, differences in recipient bed width and graft sizes for isolated and multiple GRs may be considered limitations for RC, healing, and pain assessments.

This study had a split-mouth design with both single and multiple defects. Split-mouth design was more objective than parallel design for clinical studies.^3,17^ In our view, researchers may find it difficult to provide the number of patients with both test and control defects required for statistical power of research in a split-mouth design. Regarding the power of this study, no grouping (isolated/multiple) was performed but the number of defects was analyzed. In the literature, studies generally show the results of isolated and multiple defects separately. Factors that are effective in this respect include defect, recipient bed and graft width changes, and differences in vascularization and wound healing, as well as clinical parameters such as pain reported by the patient.^38-40^The defect-related features such as RW, KTW, KTT, and the teeth were determined to be symmetrical in the same jaw for the test and control groups in each patient, and the negative effects of the study were reduced as much as possible.

## Conclusion

Within the limits of this 6-month follow-up study, CGF did not improve clinical outcomes, especially primary-outcome RC when compared with CTG. This method had a more positive effect only on postoperative pain. This finding is not enough to show the correct clinical effect of CGF against CTG with CAF, considering the defect characteristics and jaw differences in this study. Future studies should have higher standardization with large patient populations, and comparisons with other next-generation PCs, in which single and multiple defects are separately analyzed.

## References

[1] - Zucchelli G, Mounssif I. Periodontal plastic surgery. Periodontol 2000. 2015;68(1):333-68. doi: 10.1111/prd.12059. 10.1111/prd.12059 25867992

[2] - Chambrone L, Ortega MA, Sukekava F, Rotundo R, Kalemaj Z, Buti J, et al. Root coverage procedures for treating single and multiple recession-type defects: an updated Cochrane systematic review. J Periodontol. 2019. doi: 10.1002/JPER.19-0079 10.1002/JPER.19-0079 31361330

[3] - Eren G, Atilla G. Platelet-rich fibrin in the treatment of localized gingival recessions: a split-mouth randomized clinical trial. Clin Oral Investig. 2014;18(8):1941-8. doi: 10.1007/s00784-013-1170-5 10.1007/s00784-013-1170-5 24362634

[4] - Cheung WS, Griffin TJ. A comparative study of root coverage with connective tissue and platelet concentrate grafts: 8-month results. J Periodontol. 2004;75(12):1678-87. doi: 10.1902/jop.2004.75.12.1678 10.1902/jop.2004.75.12.1678 15732871

[5] - Del Fabbro M, Bortolin M, Taschieri S, Weinstein R. Is platelet concentrate advantageous for the surgical treatment of periodontal diseases? A systematic review and meta-analysis. J Periodontol. 2011;82(8):1100-11. doi: 10.1902/jop.2010.100605 10.1902/jop.2010.100605 21189090

[6] - Miron RJ, Zucchelli G, Pikos MA, Salama M, Lee S, Guillemette V, et al. Use of platelet-rich fibrin in regenerative dentistry: a systematic review. Clin Oral Investig. 2017;21(6):1913-27. doi: 10.1007/s00784-017-2133-z 10.1007/s00784-017-2133-z 28551729

[7] - Dohan Ehrenfest DM, Del Corso M, Diss A, Mouhyi J, Charrier JB. Three-dimensional architecture and cell composition of a Choukroun's platelet-rich fibrin clot and membrane. J Periodontol. 2010;81(4):546-55. doi: 10.1902/jop.2009.090531 10.1902/jop.2009.090531 20373539

[8] - Rodella LF, Favero G, Boninsegna R, Buffoli B, Labanca M, Scari G, et al. Growth factors, CD34 positive cells, and fibrin network analysis in concentrated growth factors fraction. Microsc Res Tech. 2011;74(8):772-7. doi: 10.1002/jemt.20968 10.1002/jemt.20968 21780251

[9] 9 - Yu B, Wang Z. Effect of concentrated growth factors on beagle periodontal ligament stem cells *in vitro*. Mol Med Rep. 2014; 9(1):235-42. doi: 10.3892/mmr.2013.1756 10.3892/mmr.2013.1756 24173502

[10] - Kim TH, Kim SH, Sandor GK, Kim YD. Comparison of platelet-rich plasma (PRP), platelet-rich fibrin (PRF), and concentrated growth factor (CGF) in rabbit-skull defect healing. Arch Oral Biol. 2014;59(5):550-8. doi: 10.1016/j.archoralbio.2014.02.004 10.1016/j.archoralbio.2014.02.004 24667430

[11] - Kim JM, Sohn DS, Bae MS, Moon JW, Lee JH, Park IS. Flapless transcrestal sinus augmentation using hydrodynamic piezoelectric internal sinus elevation with autologous concentrated growth factors alone. Implant Dent. 2014;23(2):168-74. doi: 10.1097/ID.0000000000000053 10.1097/ID.0000000000000053 24637529

[12] - Isler SC, Soysal F, Ceyhanlı T, Bakırarar B, Unsal B. Regenerative surgical treatment of peri-implantitis using either a collagen membrane or concentrated growth factor: a 12-month randomized clinical trial. Clin Implant Dent Relat Res. 2018;20(5):703-12. doi: 10.1111/cid.12661 10.1111/cid.12661 30118569

[13] - Isobe K, Watanebe T, Kawabata H, Kitamura Y, Okudera T, Okudera H, et al. Mechanical and degradation properties of advanced platelet-rich fibrin (A-PRF), concentrated growth factors (CGF), and platelet-poor plasma-derived fibrin (PPTF). Int J Implant Dent. 2017;3(1):17. doi: 10.1186/s40729-017-0081-7 10.1186/s40729-017-0081-7 PMC5413460 28466249

[14] - Bozkurt Doğan S, Öngöz Dede F, Balli U, Atalay EN, Durmuşlar MC. Concentrated growth factor in the treatment of adjacent multiple gingival recessions: a split-mouth randomized clinical trial. J Clin Periodontol. 2015;42(9):868-75. doi: 10.1111/jcpe.12444 10.1111/jcpe.12444 26269089

[15] - Aroca S, Keglevich T, Barbieri B, Gera I, Etienne D. Clinical evaluation of a modified coronally advanced flap alone or in combination with a platelet-rich fibrin membrane for the treatment of adjacent multiple gingival recessions: a 6-month study. J Periodontol. 2009;80(2):244-52. doi: 10.1902/jop.2009.080253 10.1902/jop.2009.080253 19186964

[16] - Loe H. The Gingival Index, the Plaque Index and the Retention Index Systems. J Periodontol. 1967;38(6):Suppl:610-6. doi: 10.1902/jop.1967.38.6.610 10.1902/jop.1967.38.6.610 5237684

[17] - Keceli HG, Kamak G, Erdemir EO, Evginer MS, Dolgun A. The adjunctive effect of platelet-rich fibrin to connective tissue graft in the treatment of buccal recession defects: results of a randomized, parallel-group controlled trial. J Periodontol. 2015;86(11):1221-30. doi: 10.1902/jop.2015.150015 10.1902/jop.2015.150015 26177630

[18] - Jankovic S, Aleksic Z, Klokkevold P, Lekovic V, Dimitrijevic B, Kenney EB, et al. Use of platelet-rich fibrin membrane following treatment of gingival recession: a randomized clinical trial. Int J Periodontics Restorative Dent. 2012;32(2):41-50. 22292152

[19] - Jankovic S, Aleksic Z, Milinkovic I, Dimitrijevic B. The coronally advanced flap in combination with platelet-rich fibrin (PRF) and enamel matrix derivative in the treatment of gingival recession: a comparative study. European J Esthet Dent. 2010;5(3):260-73. 20820456

[20] - Zucchelli G, Mele M, Mazzotti C, Marzadori M, Montebugnoli L, De Sanctis M. Coronally advanced flap with and without vertical releasing incisions for the treatment of multiple gingival recessions: a comparative controlled randomized clinical trial. J Periodontol. 2009;80(7):1083-94. doi: 10.1902/jop.2009.090041 10.1902/jop.2009.090041 19563288

[21] - Langer B, Langer L. Subepithelial connective tissue graft technique for root coverage. J Periodontol. 1985;56(12):715-20. doi: 10.1902/jop.1985.56.12.715 10.1902/jop.1985.56.12.715 3866056

[22] - Tunalı M, Özdemir H, Arabacı T, Gürbüzer B, Pikdöken L, Firatli E. Clinical evaluation of autologous platelet-rich fibrin in the treatment of multiple adjacent gingival recession defects: a 12-month study. Int J Periodontics Restorative Dent. 2015;35(1):105-14. doi: 10.11607/prd.1826 10.11607/prd.1826 25734713

[23] - Moraschini V, Barboza ES. Use of platelet-rich fibrin membrane in the treatment of gingival recession: a systematic review and meta-analysis. J Periodontol. 2016;87(3):281-90. doi: 10.1902/jop.2015.150420 10.1902/jop.2015.150420 26561997

[24] - Castro AB, Meschi N, Temmerman A, Pinto N, Lambrechts P, Teughels W, et al. Regenerative potential of leucocyte- and platelet-rich fibrin. Part A: intra-bony defects, furcation defects and periodontal plastic surgery. A systematic review and meta-analysis. J Clin Periodontol. 2017;44(1):67-82. doi: 10.1111/jcpe.12643 10.1111/jcpe.12643 PMC5248642 27783851

[25] - El Bagdadi K, Kubesch A, Yu X, Al-Maawi S, Orlowska A, Dias A, et al. Reduction of relative centrifugal forces increases growth factor release within solid platelet-rich-fibrin (PRF)-based matrices: a proof of concept of LSCC (low speed centrifugation concept). Eur J Trauma Emerg Surg. 2019;45(3):467-79. doi: 10.1007/s00068-017-0785-7 10.1007/s00068-017-0785-7 PMC6579868 28324162

[26] - Tunali M, Özdemir H, Küçükodaci Z, Akman S, Yaprak E, Toker H, et al. A novel platelet concentrate: titanium-prepared platelet-rich fibrin. BioMed Res Int. 2014;2014:209548. doi: 10.1155/2014/209548 10.1155/2014/209548 PMC3915853 24563860

[27] - Uzun BC, Ercan E, Tunali M. Effectiveness and predictability of titanium-prepared platelet-rich fibrin for the management of multiple gingival recessions. Clin Oral Investig. 2018;22(3):1345-54. 10.1007/s00784-017-2211-2 28990126

[28] - Salhi L, Lecloux G, Seidel L, Rompen E, Lambert F. Coronally advanced flap versus the pouch technique combined with a connective tissue graft to treat Miller's class I gingival recession: a randomized controlled trial. J Clin Periodontol. 2014;41(4):387-95. doi: 10.1111/jcpe.12207 10.1111/jcpe.12207 24720640

[29] - Kuka S, Ipci SD, Cakar G, Yilmaz S. Clinical evaluation of coronally advanced flap with or without platelet-rich fibrin for the treatment of multiple gingival recessions. Clin Oral Investig. 2018;22(3):1551-8. doi: 10.1007/s00784-017-2225-9 10.1007/s00784-017-2225-9 29058084

[30] - Gobbato L, Nart J, Bressan E, Mazzocco F, Paniz G, Lops D. Patient morbidity and root coverage outcomes after the application of a subepithelial connective tissue graft in combination with a coronally advanced flap or via a tunneling technique: a randomized controlled clinical trial. Clin Oral Investig. 2016;20(8):2191-202. doi: 10.1007/s00784-016-1721-7 10.1007/s00784-016-1721-7 26814715

[31] - Santamaria MP, Neves F, Silveira CA, Mathias IF, Fernandes-Dias SB, Jardini MA, et al. Connective tissue graft and tunnel or trapezoidal flap for the treatment of single maxillary gingival recessions: a randomized clinical trial. J Clin Periodontol. 2017;44(5):540-7. doi: 10.1111/jcpe.12714 10.1111/jcpe.12714 28231619

[32] - Uraz A, Sezgin Y, Yalim M, Taner IL, Çetiner D. Comparative evaluation of platelet-rich fibrin membrane and connective tissue graft in the treatment of multiple adjacent recession defects: a clinical study. J Dent Sci. 2015;10(1):36-45. doi: 10.1016/j.jds.2012.10.010

[33] - Oncu E. The use of platelet-rich fibrin versus subepithelial connective tissue graft in treatment of multiple gingival recessions: a randomized clinical trial. Int J Periodontics Restorative Dent. 2017;37(2):265-71. doi: 10.11607/prd.2741 10.11607/prd.2741 28196169

[34] - Culhaoglu R, Taner L, Guler B. Evaluation of the effect of dose-dependent platelet-rich fibrin membrane on treatment of gingival recession: a randomized, controlled clinical trial. J Appl Oral Sci. 2018;26:e20170278. doi: 10.1590/1678-7757-2017-0278 10.1590/1678-7757-2017-0278 PMC5958936 29768524

[35] - Del Corso M, Sammartino G, Dohan Ehrenfest DM. Re: “Clinical evaluation of a modified coronally advanced flap alone or in combination with a platelet-rich fibrin membrane for the treatment of adjacent multiple gingival recessions: a 6-month study”. J Periodontol. 2009;80(11):1694-7; author reply 7-9. doi: 10.1902/jop.2009.09025 10.1902/jop.2009.090253 19905939

[36] - Guiha R, el Khodeiry S, Mota L, Caffesse R. Histological evaluation of healing and revascularization of the subepithelial connective tissue graft. J Periodontol. 2001;72(4):470-8. doi: 10.1902/jop.2001.72.4.470 10.1902/jop.2001.72.4.470 11338299

[37] - Zucchelli G, Amore C, Sforza N, Montebugnoli L, De Sanctis M. Bilaminar techniques for the treatment of recession-type defects. A comparative clinical study. J Clin Periodontol. 2003;30(10):862-70. doi: 10.1034/j.1600-051x.2003.00397.x 10.1034/j.1600-051x.2003.00397.x 14710766

[38] - Zucchelli G, Mounssif I, Mazzotti C, Montebugnoli L, Sangiorgi M, Mele M, et al. Does the dimension of the graft influence patient morbidity and root coverage outcomes? A randomized controlled clinical trial. J Clin Periodontol. 2014;41(7):708-16. doi: 10.1111/jcpe.12256 10.1111/jcpe.12256 24708394

[39] - Stefanini M, Marzadori M, Aroca S, Felice P, Sangiorgi M, Zucchelli G. Decision making in root-coverage procedures for the esthetic outcome. Periodontol 2000. 2018;77(1):54-64. doi: 10.1111/prd.12205 10.1111/prd.12205 29504173

[40] 40 - Sanz M, Simion M, Working Group 3 of the European Workshop on P. Surgical techniques on periodontal plastic surgery and soft tissue regeneration: consensus report of Group 3 of the 10^th^ European Workshop on Periodontology. J Clin Periodontol. 2014;41 Suppl 15:S92-7. doi: 10.1111/jcpe.1221 10.1111/jcpe.12215 24641004

